# Cerebral aneurysm exclusion by CT angiography based on subarachnoid hemorrhage pattern: a retrospective study

**DOI:** 10.1186/1471-2377-11-8

**Published:** 2011-01-21

**Authors:** Marc Kelliny, Philippe Maeder, Stefano Binaghi, Marc Levivier, Luca Regli, Reto Meuli

**Affiliations:** 1 Department of Radiology, Centre Hospitalier Universitaire Vaudois and University of Lausanne, 1011 Lausanne, Switzerland; 2 Department of Neurosurgery, Centre Hospitalier Universitaire Vaudois and University of Lausanne, 1011 Lausanne, Switzerland; 3 Department of Neurosurgery, Rudolf Magnus Institute of Neuroscience, University Medical Center Utrecht, Netherlands

## Abstract

**Background:**

To identify patients with spontaneous subarachnoid hemorrhage for whom CT angiography alone can exclude ruptured aneurysms.

**Methods:**

An observational retrospective review was carried out of all consecutive patients with non-traumatic subarachnoid hemorrhage who underwent both CT angiography and catheter angiography to exclude an aneurysm. CT angiography negative cases (no aneurysm) were classified according to their CT hemorrhage pattern as "aneurismal", "perimesencephalic" or as "no-hemorrhage."

**Results:**

Two hundred and forty-one patients were included. A CT angiography aneurysm detection sensitivity and specificity of 96.4% and 96.0% were observed. All 35 cases of perimesencephalic or no-hemorrhage out of 78 CT angiography negatives also had negative angiography findings.

**Conclusions:**

CT angiography is self-reliant to exclude ruptured aneurysms when either a perimesencephalic hemorrhage or no-hemorrhage pattern is identified on the CT within a week of symptom onset.

## Background

Spontaneous subarachnoid hemorrhage (SAH) is a sub-type of hemorrhagic stroke with an extremely poor prognosis. Eighty-five percent of non-traumatic SAHs are caused by ruptured intracranial aneurysms. Ten percent fit into the non-aneurismal perimesencephalic hemorrhage (PMH) pattern, whose etiology remains debated. The final five percent are usually due to various rare causes, such as arteriovenous malformations [1].

When the CT is positive for subarachnoid blood, the combination of digital subtraction angiography (DSA) with 3D rotational angiography **(**3DRA) has been and is still considered the gold standard [2-6]. Meanwhile, CT angiography (CTA) has improved to a sensitivity of about 95% for detecting ruptured aneurysms, when compared to DSA [7-9]. Thus, in many centers, patients with SAH undergo CTA first which is often the basis for an endovascular or neurosurgical approach [10-12]. In any case, DSA remains a relatively riskier procedure than CTA. In patients with coiled intracranial aneurysms, the complication rate of routine angiographic surveillance is 0.43% [13] and that of general diagnostic angiography in an academic center has a complication rate of 0.3% [14].

The sensitivity of the head CT for detecting SAH is usually around 95% the first day after SAH, then gradually drops to around 58% on the fifth day [2,15-18]. The sensitivity of the head CT diminishes with less significant hemorrhage. This decreased sensitivity with time is assumed to be due to the resorption of blood during the renewal of cerebrospinal fluid several times a day.

### Non-aneurismal perimesencephalic hemorrhage

Non-aneurismal PMH has a good clinical outcome besides a low risk of re-bleeding and vasospasm. It comprises 96% of all PMHs [2,19-24]. The remaining 4% are due to vertebrobasilar aneurysms [23,25-27]. Non-aneurismal PMH is defined by a normal four-vessel high quality digital subtraction angiogram [19,28,29] combined with the following distribution of blood in the subarachnoid space:

1. The center of the hemorrhage is immediately anterior to the midbrain or the pons [19,29-31]. Variants of PMH occur in the basal cisterns around the midbrain: interpeduncular, crural, ambient, quadrigeminal, [19,29,32,33] prepontine and carotid cisterns [34].

2. Bleeding may extend up to the posterior part of the interhemispheric fissure and the basal part of the Sylvian fissure.

3. There is no intraparenchymal hematoma or frank intraventricular hemorrhage [19,29,32], although the presence of a small amount of sedimented blood in the occipital horns is acceptable.

Although non-aneurismal PMH and aneurismal hemorrhages may look alike on the CT, the clinical presentation of the first is milder with patients fully conscious or slightly disoriented. The rupture of a vein in the prepontine or interpeduncular cisterns seems to be the bleeding source of non-aneurismal PMH [35].

In patients with a benign clinical presentation and a PMH, the probability of finding a ruptured aneurysm varies between 2.5 and 8.9% [19,23,27,36,37]. Given this probability versus the clinical risks of DSA, adding DSA to a high quality CTA to exclude a ruptured aneurysm is increasingly debated [38].

### Purpose of the study

This study aims to identify when a ruptured aneurysm can be excluded by CTA, thereby avoiding catheter angiography.

## Methods

This is an observational retrospective study of all consecutive 241 adult patients who underwent both technically adequate catheter angiography and CTA for a suspicion of a ruptured aneurysm at a tertiary referral center, from January 1st 1998 to December 31st 2007. SAH was diagnosed by non-contrast CT or lumbar puncture. Arteriovenous malformations, tumors, cavernomas, infections, and trauma were excluded. Based on the final diagnosis established by conventional angiography or peroperatively, 166 patients had a bleeding aneurysm. The study, having been conducted according to the Institutional Ethical Committee's recommendations, was therefore deemed exempt of requiring its explicit approval.

### Imaging protocols

The CTA exams were performed based on a customized protocol for each CT (General Electric Healthcare, Milwaukee, Wisconsin). Table 1 shows the dates of the different CT upgrades. A timed test injection was used to determine the optimal timing of the CTA data acquisition. It consisted of a single 5 to 10 mm-thick slice (80 kVp/100 mA) positioned at the top of the frontal sinuses, acquired in a cine mode at a rate of one image every 2 s during intravenous administration of 20 mL of iodinated contrast material (2.36 mol/L {300 mg/mL} iodine) followed by 40 mL of water. The injection rate was 4-5 mL/s into an antecubital vein by means of a power injector, with a 10 s delay between the injection and the onset of data acquisition.

**Table 1:** Specifications of CT scanners from 1998 to 2007

Period	CT scanner specifications	Number of cases per scanner
1998 - November 1999	GE Highspeed Advantage CT/i **1 detector row**	39

December 1999 - April 2002	GE Lightspeed QX/i **4 detector rows**	60

May 2002 - November 2002	GE Lightspeed ultra **8 detector rows**	12

December 2002 - November 2005	GE Lightspeed 16 advantage **16 detector rows**	93

December 2005 - December 2007	GE VCT **64 detector rows**	37

GE: General Electric

The CTA data acquisition was performed in a spiral mode according to the typical parameters defined in table 2. A caudocranial scanning direction was selected, covering the volume extending from the plane situated 10 mm below the foramen magnum to the roof of the lateral ventricles. The injected volume was 50 mL with an injection rate of 4-5 mL/s followed by 40 mL of water.

**Table 2:** Typical acquisition parameters of the CT scanners

Detector rows	Rotation speed (s)	Pitch	Detector collimation (mm)	Reconstruction interval (mm)	KVp	mA
1	1.0	1.5	1.0	0.5	120	240-280

4	0.8	0.75	1.25	0.8	120	240-280

8	0.8	0.675	1.25	0.75	120	240-280

16	0.5	1.375	0.625	0.5	120	240-280

64	0.6	0.516	0.625	0.5	100	300-320

Every patient underwent four-vessel DSA via a transfemoral intra-arterial approach with multiple projections. 3DRA was done whenever deemed necessary, mainly to improve aneurysm preoperative planning [6].

### Review process

The review of the CTA and the initial reading were performed on a workstation (Advantage Windows, General Electric Healthcare, Milwaukee, Wisconsin) to allow interactive reconstruction and interpretation, which has proved to be more accurate than an isolated review of hard-copy images [39]. Axial raw images, multiplanar 2D reconstructions, maximum intensity projection reconstructions, and shaded surface display reconstructions with volume rendering technique were used for CTA review. Then, two experienced neuroradiologists blinded to the CT report and the DSA findings, independently examined the CTA of the false negative and positive cases.

Subsequently, the bleeding patterns of all CTA negative patients (true and false negatives) were classified as: perimesencephalic, aneurismal, or no visible SAH (xanthochromic lumbar puncture). This classification was based on the admission CT and the PMH definition from this article's introduction. The time between symptom onset and CT was calculated for the categories of PMH and no visible SAH.

### Data analysis

A resident supervised by neuroradiologists read the CTA first and wrote the radiological report. With respect to the catheter angiography result, as the gold standard, each case was classified with respect to the presence of an aneurysm as true positive, true negative, false positive, or false negative. The sensitivity and specificity, as well as the positive and negative predictive values regarding the detection of intracranial aneurysms, were calculated based on the values in table 3. The sensitivity was calculated with the formula True Positives/(True Positives + False Negatives), i.e. 160/(160+6) before review, 164/(164+2) after. The specificity was calculated with the formula True Negatives/(True Negatives + False Positives), i.e. 72/(72+3) before review, 75/(75+0) for the first reviewer and 74/(74+1) for the second.

**Table 3:** Results of CT angiography (CTA) vs. conventional angiography regarding the presence of an aneurysm

		Catheter Angiography	
		
		Aneurysm +	No Aneurysm -
**CTA**	**Aneurysm + (%)**	160 (66.3)	3 (1.2)
		True Positives	False Positives
	
	**No Aneurysm -(%)**	6 (2.5)	72 (30.0)
		False Negatives	True Negatives

## Results

The study sample consisted of 241 patients (105 male and 136 female) with SAH aged 20 to 86 years (mean 50.3, SD 14.2). Catheter angiographies were performed on average one day after the CTA (max. 13 days). Table 3 displays the numbers and percentages of true positive, true negative, false positive and false negative cases with respect to the presence of an aneurysm. Table 4 shows the negative CTA cases correlated with their respective hemorrhage pattern.

**Table 4:** Negative CT angiography (CTA) cases

Initial CT Hemorrhage Pattern of Negative CTA Cases	Aneurysm Found on Catheter Angiography (CTA False Negatives)	No Aneurysm Found on Catheter Angiography (CTA True Negatives)	Negative Predictive Value
**Aneurismal**	6	37	84.1%

**Perimesencephalic**	0	19	100%

**No hemorrhage**	0	16	100%

Distribution based on the initial CT and negative predictive values of the different hemorrhage groups

### Clinical presentations of the negative CTA cases

All of the patients with a perimesencephalic hemorrhage pattern presented in excellent clinical grade with a Glasgow Coma Scale (GCS) score of 15 and thus with a World Federation of Neurosurgeons (WFNS) classification score of 1. Their average hospital stay was 5 ± 3 days. Out of the 16 cases with a no-hemorrhage pattern, 13 had a GCS score of 15 and a WFNS score of 1, two had GCS scores of 13-14 and WFNS scores of 3-2 respectively. This data was unavailable for the last. The average hospital stay of patients with a no-hemorrhage pattern was 4 ± 3 days. Forty-one of the 43 patients with an aneurismal pattern had GCS scores of 12-15 and WFNS scores of 1-4. One patient had a GCS score of 3 and a WFNS score of 5. This data was unavailable for the last. The average hospital stay of patients with an aneurismal pattern was 14 ± 6 days.

There were 6 cases in which an aneurysm was missed in their original CTA readings, but each one had an aneurismal hemorrhage pattern. All 35 patients with either a PMH or no-hemorrhage pattern on the initial CT had negative findings on DSA, confirming the negative CTAs. Therefore, this sample showed a 100% negative predictive value of CTA. Thirty-three (94%) of these 35 patients had follow-up imaging. Thirty-one underwent repeat delayed DSA and two Magnetic Resonance Angiography. The last two patient's detailed neurological exams were asymptomatic at four months for one and six for the other, after the SAH.

When the blinded neuroradiologists reread the original CTAs of the false positive and false negative cases at a later date, each identified 4/6 previously missed aneurysms. The first neuroradiologist corrected all false positive cases and the second 2/3. Before review, the sensitivity and specificity were 96.4% and 96.0% respectively. Both reviewers demonstrated a 98.8% sensitivity. The first had a 100% specificity and the second 98.7%.

In this study, for the PMH patterns, the time from symptom onset to CT/CTA was between 1.3 hours and 7 days (median 19.5 hours, 3/19 cases over 48 hours) and between 2 hours and 12 days (median 25.5 hours, 7/16 cases over 48 hours) when no hemorrhage was seen.

### False negatives and false positives

**Figure 1:**
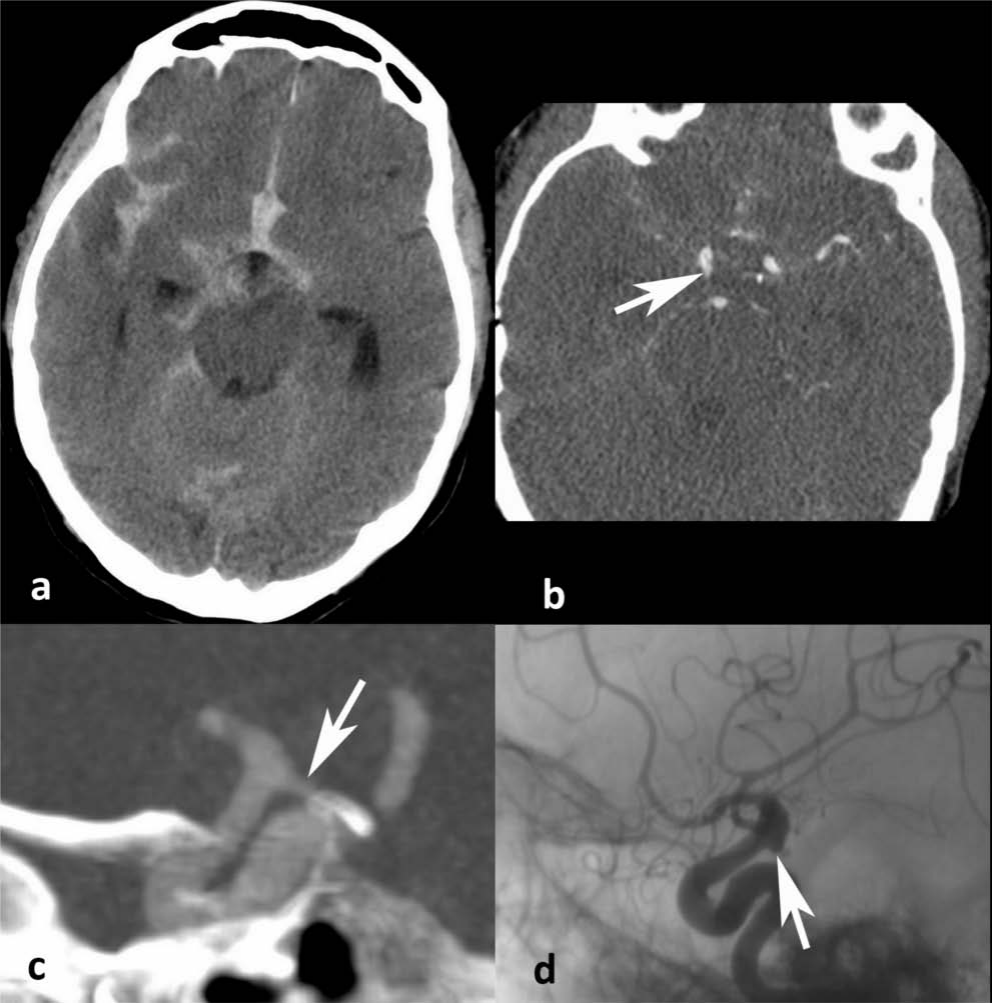
**Missed aneurysm**. Aneurysm missed by each neuroradiologist on the CT Angiography (CTA). **a **Non-contrast CT displaying the aneurismal SAH pattern. **b **CTA showing the posterior communicating aneurysm (arrow). **c **3D reconstruction of the CTA showing the aneurysm filled with contrast material, appearing to be continuous with the sphenoid bone (arrow). **d **Angiogram of the same 3 mm aneurysm on the posterior communicating artery (arrow)

Out of 6 false negatives, one aneurysm was missed by all neuroradiologists on the CTA (16-slices). It was on the posterior communicating artery, adjacent to the skull bone. This aneurysm was thus misinterpreted as being part of the sphenoid bone due to the similar density of the injected contrast agent and bone (see figure 1). DSA combined with 3DRA is an imperfect gold standard as illustrated by another case [40]. The aneurysm was found only by the third catheter angiogram, 18 days after the bleeding, whereas a reviewer recognized it on the CTA. It was partially thrombosed and located on the middle cerebral artery. Only one of the reviewers missed a third aneurysm, found on the left middle cerebral artery (16-slices). The last three false negatives were not identified on the initial CTA reading, but detected later by both reviewers. They were found on: the left anterior cerebral artery (16-slice CT), the right anterior inferior cerebellar artery (16-slice CT) and the left internal carotid artery (64-slice CT).

The false positives had either no hemorrhage visible on the initial CT (2/3) or an aneurismal hemorrhage pattern.

## Discussion

Thirty-one percent of the patients in this study sample did not have a ruptured aneurysm (19% had an aneurismal pattern, 8% had a PMH pattern, and 7% had no-hemorrhage). This distribution into different SAH categories is comparable to that of Little et al 2007. That sample consisted of 23% without a ruptured aneurysm on the initial catheter angiography (16% had an aneurismal pattern, 4% had a PMH pattern, and 3% had no-hemorrhage) [40].

The present study's sensitivity and specificity are similar to others. A blinded review of false negative and positive cases corrected at least two thirds of them, thus showing its importance. A second reading proved useful in cases with an aneurismal hemorrhage pattern since errors happened only then.

The negative predictive value of CTA varied depending on whether the non-contrast CT showed an aneurismal hemorrhage pattern, a PMH pattern or a no-hemorrhage pattern. When the non-contrast CT displayed a PMH or no-hemorrhage pattern, the CTA negative predictive value and sensitivity were both 100% with respect to 3DRA. This implied that in these cases, CTA was as good as 3DRA to exclude an aneurysm. Hence, CTA could have been used as the sole diagnostic imaging technique with its lower risks and costs and with greater facility of access (while the patient was still in the CT scanner).

First of all, Agid et al [41] retrospectively evaluated 193 CTAs with negative findings performed for SAH. Of these patients, 68 had a diffuse aneurismal or peripheral sulcal pattern, 93 had PMH and 32 had no blood on CT. All patients with PMH and with no blood on CT did not have an aneurysm detected by DSA. They concluded that in patients with SAH, negative CTA findings are reliable in ruling out aneurysms in the PMH pattern or no blood on CT. DSA is indicated in the diffuse aneurismal pattern of SAH, and repeat delayed DSA is required if the initial DSA findings are negative.

Secondly, Kershenovich et al [42] deduced from a retrospective study of 30 patients with negative CTAs, DSAs and PMH that brain CTA alone is a good and conclusive diagnostic tool to rule out aneurysms in patients presenting with the classic PMH pattern and could thus replace DSA. They also mention 8 patients diagnosed with SAH only by LP, where an initially negative CTA scan would have been sufficient to rule out an aneurysm.

Thirdly, in a series of 60 patients with SAH and negative CTA results, Westerlaan et al [10] reported 17 with a nonperimesencephalic (aneurismal) blood distribution, 30 with PMH, and 13 without blood on CT. DSA was performed in 74% of the patients with PMH and without blood on the initial CT. All were true negatives. In the aneurismal blood distribution group, an aneurysm was found in 5 (29%) patients, including 1 aneurysm seen only on DSA.

Ruigrok et al's [38] sensitivity analysis to evaluate the effect of different strategies following a PMH pattern shows that CTA only is the best diagnostic strategy. A fifth study reported a sensitivity, specificity, and negative predictive value of 100% for 15 patients with a PMH pattern [23]. It concluded that CTA was an adequate screening examination for vertebrobasilar aneurysms.

Pooling the results of these five studies with ours adds up to 187 patients with PMH and 69 patients with no-hemorrhage on CT in five different countries. These demonstrate with greater confidence the reliability of CTA as the sole imaging diagnostic tool in the context of a PMH or no blood on CT.

Furthermore, this study verifies the dependability of CTA in patients with PMH or no blood on CT, even in the face of the latest gold standard, 3DRA and follow-up imaging (94% of our cases). Besides, it adds more patients in a real life clinical setting, for a condition with a low incidence.

The variability in time between symptom onset and CT (1.3 hours to 12 days) may have changed the category of certain cases from an aneurismal hemorrhage pattern to no visible hemorrhage, due to blood resorption with time. This did not affect the present study's results. Though, applying its conclusions in other settings, an aneurysm could be missed in patients with originally an aneurismal hemorrhage pattern who do not undergo catheter angiography. Therefore, repetition of this research in several institutions is required to explore this point.

Successive generations of CT with an increased number of detectors have provided clearer images for improved diagnostic comfort according to professional experience. Otherwise, no objective improvement in accuracy was observed with the evolution of scanners, as false negatives were scattered throughout the years and models. All the false positives and more false negatives were found when the 16-slice scanner was used, for a standardized number of cases. The most likely explanation appears to be the difference in pitch (table 2) or the contribution of chance as the number of misses is small and 38.7% of all cases were done on this machine.

## Conclusions

This study shows that CT angiography is sufficient to exclude ruptured aneurysms without catheter angiography for patients with a PMH pattern or without any hemorrhage on the CT, when done within a week of symptom onset. For all other SAH cases, if the CT angiogram is negative, catheter angiography is still required.

## Competing interests

The authors declare that they have no competing interests.

## Authors' contributions

MK acquired the data, analyzed and interpreted the data, performed statistical analysis and drafted the manuscript. PM reviewed the CT angiograms, classified the subarachnoid hemorrhages and made critical revision of the manuscript for important intellectual content. SB reviewed the CT angiograms and made critical revision of the manuscript for important intellectual content. ML made critical revision of the manuscript for important intellectual content. LR acquired the data and made critical revision of the manuscript for important intellectual content. RM conceived and designed the research, acquired the data, analyzed and interpreted the data, drafted the manuscript, handled supervision, made critical revision of the manuscript for important intellectual content. All authors read and approved the final manuscript.

## Pre-publication history

The pre-publication history for this paper can be accessed here:

http://www.biomedcentral.com/1471-2377/11/8/prepub

## References

[B1] van Gijn J Kerr RS Rinkel GJ Subarachnoid haemorrhage Lancet 2007 369 306 318 10.1016/S0140-6736(07)60153-6 17258671

[B2] van der Wee N Rinkel GJ Hasan D van Gijn J Detection of subarachnoid haemorrhage on early CT: Is lumbar puncture still needed after a negative scan? J Neurol Neurosurg Psychiatry 1995 58 357 359 10.1136/jnnp.58.3.357 7897421 PMC1073376

[B3] van Rooij WJ Peluso JP Sluzewski M Beute GN Additional value of 3D rotational angiography in angiographically negative aneurismal subarachnoid hemorrhage: how negative is negative? AJNR Am J Neuroradiol 2008 29 962 966 10.3174/ajnr.A0972 18258701 PMC8128569

[B4] van Rooij WJ Sprengers ME de Gast AN Peluso JP Sluzewski M 3D rotational angiography: the new gold standard in the detection of additional intracranial aneurysms AJNR Am J Neuroradiol 2008 29 976 979 10.3174/ajnr.A0964 18258703 PMC8128578

[B5] Hirai T Korogi Y Suginohara K Ono K Nishi T Uemura S Yamura M Yamashita Y Clinical usefulness of unsubtracted 3D digital angiography compared with rotational digital angiography in the pretreatment evaluation of intracranial aneurysms AJNR Am J Neuroradiol 2003 24 1067 1074 12812928 PMC8149013

[B6] Hochmuth A Spetzger U Schumacher M Comparison of three-dimensional rotational angiography with digital subtraction angiography in the assessment of ruptured cerebral aneurysms AJNR Am J Neuroradiol 2002 23 1199 1205 12169480 PMC8185715

[B7] Chappell ET Moure FC Good MC Comparison of computed tomographic angiography with digital subtraction angiography in the diagnosis of cerebral aneurysms: a meta-analysis Neurosurgery 2003 52 624 631 10.1227/01.NEU.0000047895.82857.EB 12590688

[B8] Wintermark M Uske A Chalaron M Regli L Maeder P Meuli R Schnyder P Binaghi S Multislice computerized tomography angiography in the evaluation of intracranial aneurysms: a comparison with intraarterial digital subtraction angiography J Neurosurg 2003 98 828 836 10.3171/jns.2003.98.4.0828 12691409

[B9] Villablanca JP Hooshi P Martin N Jahan R Duckwiler G Lim S Frazee J Gobin YP Sayre J Bentson J Viñuela F Three-dimensional helical computerized tomography angiography in the diagnosis, characterization and management of middle cerebral artery aneurysms: comparison with conventional angiography and intraoperative findings J Neurosurg 2002 97 1322 1332 10.3171/jns.2002.97.6.1322 12507130

[B10] Westerlaan HE Gravendeel J Fiore D Metzemaekers JD Groen RJ Mooij JJ Oudkerk M Multislice CT angiography in the selection of patients with ruptured intracranial aneurysms suitable for clipping or coiling Neuroradiology 2007 49 997 1007 10.1007/s00234-007-0293-2 17891387 PMC2082066

[B11] Lubicz B Levivier M François O François O Thoma P Sadeghi N Collignon L Balériaux D Sixty-four-row multisection CT angiography for detection and evaluation of ruptured intracranial aneurysms: interobserver and intertechnique reproducibility AJNR Am J Neuroradiol 2007 28 1949 1955 10.3174/ajnr.A0699 17898200 PMC8134231

[B12] Agid R Lee SK Willinsky RA Farb RI terBrugge KG Acute subarachnoid hemorrhage: using 64-slice multidetector CT angiography to "triage" patients' treatment Neuroradiology 2006 48 787 794 10.1007/s00234-006-0129-5 17009025

[B13] Ringer AJ Rodriguez-Mercado R Veznedaroglu E Levy EI Hanel RA Mericle RA Lopes DK Lanzino G Boulos AS Defining the risk of retreatment for aneurysm recurrence or residual after initial treatment by endovascular coiling: a multicenter study Neurosurgery 2009 65 311 315 10.1227/01.NEU.0000349922.05350.96 19625910

[B14] Fifi JT Meyers PM Lavine SD Cox V Silverberg L Mangla S Pile-Spellman J Complications of modern diagnostic cerebral angiography in an academic medical center J Vasc Interv Radiol 2009 20 442 447 10.1016/j.jvir.2009.01.012 19246211

[B15] Kassell NF Torner JC Haley EC Jr Jane JA Adams HP Kongable GL The International Cooperative Study on the timing of aneurysm surgery, I: Overall management results J Neurosurg 1990 73 18 10.3171/jns.1990.73.1.0018 2191090

[B16] Sames TA Storrow AB Finkelstein JA Magoon MR Sensitivity of new-generation computed tomography in subarachnoid hemorrhage Acad Emerg Med 1996 3 16 10.1111/j.1553-2712.1996.tb03296.x 8749962

[B17] Sidman R Connolly E Lemke T Subarachnoid hemorrhage diagnosis: lumbar puncture is still needed when the computed tomography scan is normal Acad Emerg Med 1996 3 827 10.1111/j.1553-2712.1996.tb03526.x 8870753

[B18] Boesiger BM Shiber JR Subarachnoid hemorrhage diagnosis by computed tomography and lumbar puncture: are fifth generation CT scanners better at identifying subarachnoid hemorrhage? J Emerg Med 2005 29 23 27 10.1016/j.jemermed.2005.02.002 15961003

[B19] Rinkel GJ Wijdicks EF Vermeulen M Ramos LM Tanghe HL Hasan D Meiners LC van Gijn J Nonaneurysmal perimesencephalic subarachnoid hemorrhage: CT and MR patterns that differ from aneurysm rupture AJNR Am J Neuroradiol 1991 12 829 834 1950905 PMC8333493

[B20] Rinkel GJ Wijdicks EF Vermeulen M Hasan D Brouwers PJ van Gijn J The clinical course of perimesencephalic nonaneurysmal subarachnoid hemorrhage Ann Neurol 1991 29 463 468 10.1002/ana.410290503 1859176

[B21] Rinkel GJ Wijdicks EF Hasan D Kienstra GE Franke CL Hageman LM Vermeulen M van Gijn J Outcome in patients with subarachnoid haemorrhage and negative angiography according to pattern of haemorrhage on computed tomography Lancet 1991 338 964 968 10.1016/0140-6736(91)91836-J 1681340

[B22] Brilstra EH Hop JW Rinkel GJ Quality of life after perimesencephalic haemorrhage J Neurol Neurosurg Psychiatry 1997 63 382 384 10.1136/jnnp.63.3.382 9328259 PMC2169713

[B23] Velthuis BK Rinkel GJ Ramos LM Witkamp TD van Leeuwen MS Perimesencephalic hemorrhage. Exclusion of vertebrobasilar aneurysms with CT angiography Stroke 1999 30 1780 1786 10229751 10.1161/01.str.30.5.1103

[B24] Ildan F Tuna M Erman T Gocer AI Cetinalp E Prognosis and prognostic factors in nonaneurysmal perimesencephalic hemorrhage: A follow-up study in 29 patients Surg Neurol 2002 57 160 166 10.1016/S0090-3019(02)00630-4 12009538

[B25] Goergen SK Barrie D Sacharias N Waugh JR Perimesencephalic subarachnoid haemorrhage: negative angiography and favourable prognosis Australas Radiol 1993 37 156 160 10.1111/j.1440-1673.1993.tb00040.x 8512504

[B26] Kitahara T Ohwada T Tokiwa K Kurata A Miyasaka Y Yada K Kan S Clinical study in patients with perimesencephalic subarachnoid hemorrhage of unknown etiology No Shinkei Geka 1993 21 903 908 8413803

[B27] Pinto AN Ferro JM Canhao P Campos J How often is a perimesencephalic subarachnoid haemorrhage CT pattern caused by ruptured aneurysms? Acta Neurochir (Wien) 1993 124 79 81 10.1007/BF01401126 8304074

[B28] Ausman JI Perimesencephalic nonaneurysmal subarachnoid hemorrhage: what is it? what are we missing? Surg Neurol 2002 57 211 10.1016/S0090-3019(02)00634-1 12009557

[B29] Schwartz TH Solomon RA Perimesencephalic nonaneurysmal subarachnoid hemorrhage: review of the literature Neurosurgery 1996 39 433 440 10.1097/00006123-199609000-00001 8875472

[B30] Zentner J Solymosi L Lorenz M Subarachnoid hemorrhage of unknown etiology Neurol Res 1996 18 220 226 8837056 10.1080/01616412.1996.11740408

[B31] Schievink WI Wijdicks EF Pretruncal subarachnoid hemorrhage: an anatomically correct description of the perimesencephalic subarachnoid hemorrhage Stroke 1997 28 2572 9412654

[B32] van Gijn J van Dongen KJ Vermeulen M Hijdra A Perimesencephalic hemorrhage: a nonaneurysmal and benign form of subarachnoid hemorrhage Neurology 1985 35 493 497 3982634 10.1212/wnl.35.4.493

[B33] Schwartz TH Farkas J Quadrigeminal non-aneurysmal subarachnoid hemorrhage: a true variant of perimesencephalic subarachnoid hemorrhage: case report Clin Neurol Neurosurg 2003 105 95 98 10.1016/S0303-8467(02)00112-9 12691799

[B34] Schwartz TH Mayer SA Quadrigeminal variant of perimesencephalic nonaneurysmal subarachnoid hemorrhage Neurosurgery 2000 46 584 588 10.1097/00006123-200003000-00012 10719854

[B35] van der Schaaf I Velthuis BK Gouw A Rinkel GJ Venous drainage in perimesencephalic hemorrhage Stroke 2004 35 1614 1618 10.1161/01.STR.0000131657.08655.ce 15166390

[B36] van Calenbergh F Plets C Goffin J Velghe L Nonaneurysmal subarachnoid hemorrhage: prevalence of perimesencephalic hemorrhage in a consecutive series Surg Neurol 1993 39 320 323 10.1016/0090-3019(93)90014-R 8488453

[B37] Alén JF Lagares A Lobato RD Gómez PA Rivas JJ Ramos A Comparison between perimesencephalic nonaneurysmal subarachnoid hemorrhage and subarachnoid hemorrhage caused by posterior circulation aneurysms J Neurosurg 2003 98 529 535 12650424 10.3171/jns.2003.98.3.0529

[B38] Ruigrok YM Rinkel GJ Buskens E Velthuis BK van Gijn J Perimesencephalic hemorrhage and CT angiography: a decision analysis Stroke 2000 31 2976 2983 11108759 10.1161/01.str.31.12.2976

[B39] Young N Dorsch NW Kingston RJ Soo MY Robinson A Spiral CT scanning in the detection and evaluation of aneurysms of the Circle of Willis Surg Neurol 1998 50 50 61 10.1016/S0090-3019(98)00015-9 9657493

[B40] Little AS Garrett M Germain R Farhataziz N Albuquerque FC McDougall CG Zabramski JM Nakaji P Spetzler RF Evaluation of patients with spontaneous subarachnoid hemorrhage and negative angiography Neurosurgery 2007 61 1139 1150 10.1227/01.neu.0000306091.30517.e7 18162892

[B41] Agid R Andersson T Almqvist H Willinsky RA Lee SK terBrugge KG Farb RI Söderman M Negative CT angiography findings in patients with spontaneous subarachnoid hemorrhage: when is digital subtraction angiography still needed? AJNR Am J Neuroradiol 2010 31 696 705 10.3174/ajnr.A1884 19942709 PMC7964209

[B42] Kershenovich A Rappaport ZH Maimon S Brain computed tomography angiographic scans as the sole diagnostic examination for excluding aneurysms in patients with perimesencephalic subarachnoid hemorrhage Neurosurgery 2006 59 798 802 10.1227/01.NEU.0000232724.19888.C6 16915122

